# Nasal Irrigation in Children: From Pathophysiological Rationale to Clinical Practice

**DOI:** 10.3390/children13070851

**Published:** 2026-06-24

**Authors:** Luca Pecoraro, Andrea Dell’Anna, Elisabetta Di Muri, Emiliano Altavilla, Francesca Marasciulo, Alessio Signore, Flavia Indrio

**Affiliations:** 1 Department of Experimental Medicine, Pediatric Section, University of Salento Hospital “Vito Fazzi”, 73100 Lecce, Italy; 2 Pediatric Unit, Ospedale Vito Fazzi, ASL Lecce, 73100 Lecce, Italy

**Keywords:** nasal irrigation, mucociliary clearance, allergic rhinitis, upper respiratory tract infections

## Abstract

**Highlights:**

**What are the main findings?**

- Nasal irrigation supports mucociliary clearance by improving mucus hydration, facilitating the removal of pathogens, allergens, pollutants, and inflammatory mediators from the nasal cavity.
- Current evidence suggests that nasal irrigation can represent a safe and well-tolerated adjunctive intervention that may improve symptoms in common pediatric upper airway disorders.

**What are the implication of the main findings?**

- Integrating the pathophysiology of mucociliary dysfunction, environmental exposure, and pediatric airway characteristics provides a comprehensive framework for understanding the clinical role of nasal irrigation in children.
- Appropriate technique, age-specific administration, caregiver education, and correct solution selection are essential to maximize clinical benefits and support rational, non-pharmacological management of pediatric respiratory diseases.

**Abstract:**

Upper respiratory tract infections and inflammatory nasal disorders are highly prevalent in childhood and represent a major cause of morbidity and healthcare utilization. Humans are continuously exposed to airborne microorganisms, allergens, and pollutants. Although the nasal mucosa provides effective mechanical and immunological defenses, these mechanisms may be impaired by inflammation, environmental pollutants, and mucociliary dysfunction, increasing susceptibility to infection and airway inflammation. Nasal irrigation (NI) contributes to the restoration of nasal homeostasis by mechanically removing mucus, pathogens, allergens, and inflammatory mediators, while also improving mucociliary clearance (MC), mucus rheology, and epithelial barrier function. Hypertonic solutions (HS) may provide additional osmotic and decongestant effects. Current evidence suggests that NI is a safe and well-tolerated adjunctive intervention that may improve symptoms and support mucosal function in acute and chronic upper airway diseases. This narrative review provides an updated overview of NI, with particular focus on pediatric populations. This paper integrates the pathophysiological mechanisms of mucociliary dysfunction, environmental exposures, and pediatric-specific anatomical and functional characteristics into a unified framework to understand the role of NI in childhood respiratory diseases. Clinical indications, administration techniques, solution selection, safety aspects, and age-specific practical considerations are discussed, highlighting the importance of appropriate technique, caregiver education, and adherence to basic hygiene principles.

## 1. Introduction

Upper respiratory tract infections (URTIs) are among the leading causes of morbidity in children and represent a frequent reason for outpatient consultations. In early life, high exposure to respiratory pathogens, combined with the immaturity of the immune system, results in a high incidence of infectious episodes, with a significant impact on both the child’s and the family’s quality of life, as well as on healthcare resource utilization [1,2,3]. Since most of these infections are viral, management is mainly symptomatic. In this context, there has been increasing interest in non-pharmacological interventions aimed at improving symptom control and reducing inappropriate drug use, particularly antibiotics [4,5,6]. Nasal irrigation (NI) is one of the oldest methods used for upper airway cleansing. Originating in ancient India, it has been practiced for thousands of years and introduced into Western medicine in the early 20th century [7,8]. Despite its long-standing use, its clinical value in contemporary medical practice remains a topic of ongoing discussion [9]. In daily life, individuals are continuously exposed to numerous potentially pathogenic agents. The nasal cavity represents the primary entry point for respiratory viruses and bacteria. Environmental studies have shown that microbial concentrations differ between indoor and outdoor environments, enabling the estimation of daily inhaled particle loads under physiological conditions [10,11,12]. An individual at rest inhales approximately 8 litres of air per minute, corresponding to more than 11 cubic metres per day, and is exposed to hundreds of thousands of bacteria and a comparable number of viral particles. In addition, inhaled air contains particles from pollutants, which may exert harmful local and systemic effects [13,14]. Despite this constant exposure, the respiratory system relies on highly efficient anatomical and functional defense mechanisms. The upper airway plays a key role through the mucociliary system, which facilitates clearance of inhaled particles [12]. The nasal cavity is lined with ciliated epithelium and, in older individuals, with nasal hairs that filter larger particles. The turbinates, covered by mucus, contribute to particle retention, while airflow dynamics promote deposition in the nasopharynx. Lymphoid structures such as the adenoids and tonsils also contribute to local immune defense [15,16]. However, these protective mechanisms may be overwhelmed under certain conditions, such as high microbial exposure, increased pathogen virulence, or enhanced microorganism adherence to and colonization of the mucosa [17,18]. In particular, impairment of nasal mucosal integrity and function is a key factor associated with increased susceptibility to infection [19]. Within this framework, NI has been proposed as a supportive measure. By mechanically removing mucus, pathogens, allergens, and inhaled particles, it may help preserve mucosal function and enhance MC [20,21]. This may result in reduced local microbial burden and a less favorable environment for viral and bacterial proliferation [22]. Despite its widespread clinical use and perception as a safe and simple intervention, the available evidence remains heterogeneous, both regarding its efficacy and the optimal administration techniques, particularly in pediatric populations [22,23]. Accordingly, this narrative review aims to provide an updated overview of NI by integrating the pathophysiological mechanisms of mucociliary dysfunction, environmental exposure, and pediatric-specific anatomical and functional characteristics into a unified framework for understanding its role in childhood upper airway diseases. In addition, the available clinical evidence, practical administration techniques, safety aspects, and age-related considerations are discussed.

## 2. Literature Search Strategy

A non-systematic literature search was performed in PubMed and Scopus to identify relevant studies on nasal irrigation, mucociliary clearance, upper respiratory tract infections, rhinosinusitis, allergic rhinitis, and pediatric respiratory diseases. The search strategy employed the following primary keywords: “nasal irrigation”, “mucociliary clearance”, “upper respiratory tract infections”, “rhinosinusitis”, “allergic rhinitis”, and “pediatric respiratory diseases”. To optimize data retrieval, the search terms were expanded to include secondary keywords extracted from the investigated pathophysiological and therapeutic targets, such as: “isotonic saline”, “hypertonic saline”, “nasal spray”, “mucociliary dysfunction”, “environmental exposure”, and “airway inflammation”. Priority was given to randomized controlled trials, systematic reviews, meta-analyses, and international guidelines published in English. Case reports, expert opinions, and non-English publications were excluded. Additional articles were identified through manual screening of reference lists. The aim of this narrative review was to provide an integrated overview of current evidence and pathophysiological concepts rather than a systematic synthesis of the literature about NI with a focus on pediatric age.

## 3. Mucociliary Clearance: Pathophysiological Considerations

MC is one of the principal innate defense mechanisms of the respiratory system and plays a central role in maintaining airway homeostasis [24,25,26]. It consists of an integrated system involving the ciliated respiratory epithelium, the overlying mucus layer, and coordinated physical and biochemical processes that facilitate the removal of inhaled particles, microorganisms, and cellular debris [25,27]. The epithelium of the upper airways is composed of ciliated and mucus-secreting cells, primarily goblet cells, organized into a biphasic mucus system [28]. This system includes a lower, low-viscosity sol layer that permits ciliary motion and an overlying gel layer with higher viscosity that traps inhaled particles and pathogens [29]. Coordinated ciliary beating, occurring at a frequency of approximately 10–15 Hz, propels the mucus layer towards the nasopharynx, where it is subsequently swallowed or expelled [30]. The effectiveness of MC depends on a delicate equilibrium between mucus production, its rheological properties, and ciliary function. Alterations in any of these components may impair clearance efficiency [27,31]. In particular, increased mucus viscosity, dehydration of the periciliary layer, or reduced ciliary motility impair mucociliary transport, favoring secretion retention and microbial proliferation [32,33]. In pediatric patients, mucociliary function presents specific physiological characteristics. In infants and young children, the airways are anatomically narrower, and MC may be less efficient due to the functional immaturity of the respiratory epithelium and reduced ability to actively clear secretions [29,34,35]. In this age group, common conditions such as viral URTIs and exposure to environmental pollutants may further impair ciliary function and increase mucus production [36]. Acute respiratory infections can directly damage the ciliated epithelium, reduce ciliary beat frequency, and alter mucus rheology, making it more viscous and less effectively transported [29,37]. This creates a self-perpetuating cycle characterized by impaired clearance, secretion accumulation, and prolonged pathogen contact with the mucosa [25]. In addition, environmental factors play a key role in impairing MC by altering the structure and function of the respiratory epithelium [38,39]. Among these, exposure to tobacco smoke, including passive smoking, represents one of the most extensively studied and clinically relevant risk factors. Even in non-smokers, secondhand smoke exposure has been associated with a measurable reduction in nasal MC, highlighting its impact even at low exposure levels [40]. The underlying pathophysiological mechanisms are multifactorial. Toxic compounds contained in tobacco smoke, such as formaldehyde, acrolein, and phenolic derivatives, exert a direct ciliotoxic effect, leading to reduced ciliary beat frequency and structural damage to ciliated epithelial cells. In parallel, smoke-induced oxidative stress promotes the generation of reactive oxygen species, which further contribute to epithelial injury and functional impairment of ciliary activity [39]. In addition, exposure to tobacco smoke alters mucus rheology by increasing its production and viscosity, thereby hindering effective mucociliary transport. Chronic exposure is also associated with persistent low-grade inflammation, characterized by cytokine release and epithelial remodeling, which further compromises mucosal defense mechanisms [39,40]. Beyond tobacco smoke, air pollution represents another major determinant of mucociliary dysfunction. Exposure to particulate matter (PM2.5 and PM10) and gaseous pollutants such as nitrogen dioxide and sulfur dioxide has been shown to induce oxidative stress, epithelial inflammation, and direct ciliary injury, ultimately resulting in reduced mucociliary efficiency [41,42]. Similarly, indoor pollutants, including biomass combustion products and volatile organic compounds, may contribute to airway irritation, increased mucus production, and impaired mucosal clearance [43]. Allergen exposure can also indirectly affect mucociliary function by inducing chronic airway inflammation, goblet cell hyperplasia, and mucus hypersecretion, thereby increasing the mucus burden and reducing clearance efficiency. Furthermore, recurrent exposure to infectious agents and airway irritants may cause transient or persistent epithelial damage, leading to reduced ciliary density and coordinated activity. Overall, these environmental factors converge in disrupting the delicate balance of the mucociliary system, thereby increasing susceptibility to respiratory infections and contributing to persistent airway dysfunction [44,45]. Within this pathophysiological framework, interventions aimed at restoring optimal mucociliary function may play a relevant role in both the prevention and management of upper respiratory tract disorders. NI with saline solutions primarily promotes mucus hydration, reduces viscosity, and facilitates mechanical clearance of secretions, thereby supporting the restoration of mucociliary function [46,47]. The rationale for NI in pediatric patients is therefore based on its ability to act on the main determinants of MC and on the homeostasis of the nasal environment [48]. Saline irrigation exerts a mechanical cleansing effect by removing mucus, cellular debris, inhaled particles, and microorganisms from the nasal cavities [48]. This reduces secretion stasis and limits the duration of pathogen contact with the nasal mucosa [49]. In addition, saline solutions improve the rheological properties of mucus by hydrating the periciliary layer, reducing mucus viscosity, and restoring optimal conditions for ciliary activity [49,50]. Through these mechanisms, NI may enhance mucociliary transport and potentially counteract some of the pathophysiological processes involved in upper airway infections [21,46]. Although it does not exert a direct antimicrobial effect, NI may also help reduce local microbial load by mechanically removing viruses and bacteria from the mucosal surface, thereby interfering with early colonization and adhesion processes [21,51,52]. This effect may be particularly relevant in the early stages of viral infections and in conditions characterized by excessive mucus production [53]. Additional, though less well-defined, effects include potential modulation of the local inflammatory response. Some studies suggest that saline irrigation may reduce mucosal edema and dilute inflammatory mediators, thereby improving nasal patency [54,55,56]. Furthermore, a potential otologic benefit of NI can be considered. Specifically, they are mediated indirectly at the nasopharynx, where the Eustachian tube (ET) orifice resides. In this context, mechanically removing mucus, crusts, and inflammatory secretions reduces periostial edema and localized inflammation around the ET opening [57,58]. This reduces functional obstruction, facilitating physiologic ET opening during swallowing or yawning, thereby improving middle-ear ventilation and pressure regulation. This framework adds a pivotal mechanism to the discussion, directly linking nasopharyngeal mucosal hygiene to the mitigation of ET dysfunction and the pathophysiology of middle-ear disease [49,59]. In pediatric populations, these mechanisms are particularly relevant due to reduced ability to clear nasal secretions independently, smaller airway caliber, and increased susceptibility to respiratory infections. In this context, NI represents a simple, safe, and well-tolerated intervention that supports physiological defense mechanisms, contributing to symptom control and improved respiratory comfort [46,60]. Overall, the rationale for NI is based on the combined mechanical, physical, and functional effects that act synergistically to restore nasal mucosal balance and counteract the pathophysiological mechanisms underlying upper respiratory tract diseases [61].

## 4. Clinical Use of NI: Indications, Technique, Solutions, and Safety

NI is widely used as an adjunctive treatment in upper respiratory conditions. Its main indication is the management of acute URTIs, where it contributes to symptom relief by reducing nasal congestion and facilitating the clearance of secretions [51,57]. It is also commonly used in acute and chronic rhinosinusitis and allergic rhinitis (AR), where it supports mucociliary function and promotes the removal of allergens and inflammatory mediators [20,58]. Preventive use has been proposed in individuals with recurrent infections, although supporting evidence remains limited [59]. The effectiveness of NI depends largely on correct technique. The procedure should be adapted to the patient’s age, clinical condition, and level of cooperation to ensure adequate cleansing while minimizing discomfort and potential complications [62]. In younger children, who are unable to clear nasal secretions effectively, irrigation is typically performed with the child in a supine or slightly inclined position, with the head turned to one side. The solution is gently instilled into one nostril using single-dose vials or needle-free syringes, allowing it to pass through the nasal cavity and exit from the opposite nostril or into the nasopharynx. Adequate volumes are required for effective clearance, although administration should be gradual to maintain tolerability. In selected cases, particularly when abundant secretions are present, gentle suction may be used as an adjunct [63]. Nasal drops and sprays are categorized as low-volume methods of NI, primarily used when the large-volume, positive-pressure systems preferred for older children are not feasible. These devices are the most common tools for neonates, infants, and toddlers who cannot tolerate compressive douching systems [46]. In older children, irrigation can be performed in a sitting or standing position, with the head slightly tilted forward and rotated. Positive-pressure devices, such as squeeze bottles, may improve the distribution of the solution within the nasal cavities. Cooperation can be enhanced by encouraging calm oral breathing during the procedure, reducing the risk of aspiration [64]. Volume is a key determinant of efficacy. Available evidence suggests that medium- to high-volume irrigation may provide greater cleansing efficiency than low-volume instillation, as it ensures more complete removal of secretions and adherent particles. However, volumes should be adjusted according to age and tolerability [65,66]. Frequency of administration varies with clinical context: during acute illness, irrigation may be performed one to three times daily, whereas lower frequencies are usually sufficient for maintenance or preventive use [67]. The literature indicates a lack of clear high-level evidence regarding the optimal frequency and duration of NI. Excessive or improper use may cause local adverse events via plausible mechanisms: high-volume, high-pressure, or HS can induce mucosal trauma, irritation, epistaxis, and ET dysfunction [49]. Meanwhile, frequent administration, particularly with HS, can cause dehydration and mucosal irritation via an osmotic effect when edema is absent or when administered long-term [68]. Caregiver education is essential, particularly in younger patients. Clear instructions on technique, volume, and safety precautions are necessary to ensure correct administration and minimize errors [69]. The choice of device also influences outcomes, as different systems vary in the volumes and pressures they deliver. Low-volume, low-pressure devices are generally preferred in younger children, whereas older patients may benefit from devices that allow more effective irrigation [70,71]. The type of saline solution plays an important role in both efficacy and tolerability. Isotonic solutions (0.9% sodium chloride) are most used, particularly in younger children, due to their good tolerability. Their main effect is mechanical, promoting the removal of mucus, debris, and pathogens while maintaining mucosal hydration and supporting mucociliary function [22,72]. HS (typically 3%) exerts an additional osmotic effect, drawing water into the airway lumen and reducing mucosal edema, thereby improving nasal patency. This may be particularly useful under conditions characterized by marked congestion. While both isotonic and HS are effective, some evidence suggests a modest advantage of hypertonic formulations in improving MC and reducing symptoms, albeit at the cost of lower tolerability, including nasal irritation or burning sensations. Irrigation volume and delivery methods further influence outcomes. Regardless of solution type, higher-volume irrigation is generally more effective than low-volume sprays, as it allows more thorough cleansing of the nasal cavities and nasopharynx [47]. Formulations enriched with additional components, such as hyaluronic acid, are available, but current evidence does not clearly demonstrate a significant clinical advantage over standard saline solutions in pediatric populations [68,73]. When performed correctly, NI has an excellent safety profile. Adverse effects are usually mild and transient, including nasal discomfort, burning sensation, sneezing, or occasional coughing. These effects typically resolve spontaneously and do not require discontinuation of treatment. Safety depends not only on technique but also on the quality of the solution [74]. The use of non-sterile water or improperly prepared solutions carries a risk of contamination and infection. Sterile, ready-to-use preparations are therefore recommended, or, as an alternative, solutions prepared using previously boiled and cooled water under appropriate hygienic conditions [75,76]. Irrigation devices should be thoroughly cleaned and dried after each use and replaced regularly. In younger children, the procedure should always be performed under adult supervision. Contraindications are uncommon but should be considered. These include significant epistaxis, acute middle ear disease, marked anatomical abnormalities of the nasal cavities, and known hypersensitivity to solution components. In such cases, use should be evaluated on an individual basis. A Delphi survey identified osteomeningeal fracture with cerebrospinal fluid leakage as an absolute contraindication and ineffective cough as a relative contraindication [77]. Swallowing disorders were also considered a potential absolute contraindication by a majority of experts [78]. Conversely, conditions such as laryngomalacia and respiratory failure were not considered contraindications, although caution was advised in cases of acute otitis media and epistaxis [77]. In adults, additional contraindications include incompletely healed facial trauma and conditions associated with increased aspiration risk, such as significant neurological or neuromuscular disorders [79]. Saline NI is widely employed to reduce nasal congestion and mucopurulent secretions, stimulate cleansing of the nasal and paranasal cavities, and promote restoration of MC [58,80]. A pilot study evaluated the effects of saline nasal irrigation on nasal cytology in 66 children with allergic rhinitis. Treatment was associated with a reduction in neutrophilic and eosinophilic inflammatory infiltrates, suggesting a potential anti-inflammatory effect of nasal irrigation [80]. NI represents a simple, safe, and low-cost adjunct in the management of upper respiratory tract conditions. Its clinical benefit is closely linked to appropriate technique, correct patient selection, and adherence to basic hygiene and safety principles. A relevant practical consideration concerns the distinction between NI and low-volume saline delivery systems, such as nasal sprays. Although both approaches employ isotonic or hypertonic saline solutions, they differ substantially in terms of volume, pressure, and distribution within the nasal cavities [65,66]. Nasal sprays typically deliver small volumes of solution and are primarily intended to moisturize the nasal mucosa and provide symptomatic relief. In contrast, NI involves the administration of larger volumes, often under positive pressure, allowing a more extensive mechanical clearance of mucus, inhaled particles, and inflammatory mediators [62,81]. Despite these theoretical and physiological differences, direct comparative evidence between NI and nasal sprays is limited [46]. To date, no high-quality randomized controlled trials have specifically compared the two modalities in pediatric populations. As a result, most available data derive from indirect comparisons, physiological studies, or trials evaluating each intervention separately [81]. From a mechanistic perspective, higher-volume irrigation is likely to provide a more effective cleansing effect and greater impact on MC [62], particularly in conditions characterized by significant secretion burden, such as acute URTIs and rhinosinusitis. Conversely, nasal sprays may be better tolerated, especially in younger children, and may be a practical option for mild symptoms or when frequent administration is required. Overall, while both modalities are safe and widely used, their roles should be considered complementary rather than interchangeable [46]. Middle-ear symptoms during or after NI—including ear pain, pressure, and discomfort plausibly related to transient ET dysfunction—are described in the literature as relevant “minor” adverse effects, particularly with high-volume or forceful delivery [60]. Similarly, a chronic rhinosinusitis guideline on nasal irrigation addresses harms and notes otalgia/ET-related issues as possible complications, reinforcing that counseling should include discussion of middle-ear symptoms and technique-related risk mitigation (e.g., avoiding excessive pressure, inappropriate device use) [49]. NI may be preferred when the therapeutic goal is active clearance of secretions and support of mucociliary function, whereas nasal sprays may be more appropriate for maintenance therapy or when tolerability and ease of use are priorities. However, further well-designed comparative studies are needed to better define the relative efficacy of these approaches.

## 5. The Peculiarity of Pediatric Age

Clinical evidence on the effectiveness of NI in pediatric populations has increased in recent years, although the available data remains heterogeneous in terms of study design, patient populations, and intervention protocols. Most evidence derives from randomized controlled trials and systematic reviews involving children with URTIs, rhinosinusitis, and AR. In acute URTIs, NI with saline solutions has been associated with improvements in key symptoms, particularly nasal congestion and rhinorrhea, and a reduction in disease duration [22,23]. Systematic reviews generally report improvements in symptom severity and clinical recovery, although study heterogeneity limits the strength of these conclusions. Similarly, meta-analyses of randomized controlled trials in infants and children have shown significant improvements in nasal symptoms and a reduced need for medications, including antibiotics, in patients treated with saline irrigation compared with controls [21,82]. In pediatric rhinosinusitis, current evidence generally favors the use of NI as adjunctive therapy. Studies have demonstrated improvements in symptom burden and quality of life in both acute and chronic forms, with a favorable safety profile [83]. In AR, saline irrigation has been associated with reductions in symptom severity and decreased reliance on pharmacological treatments, likely by removing allergens and enhancing MC [20]. Nevertheless, the widespread use of NI, together with its favorable safety profile, low cost, and good tolerability, supports its role as an adjunctive intervention in the management of common upper respiratory tract conditions in children. In early childhood, the effectiveness of nasal sprays may be limited by anatomical and functional characteristics of the nasal cavity [84]. In children under approximately 5 years of age, the nasal passages are proportionally narrower, with higher airflow resistance and a more complex anatomical configuration relative to total airway size. These factors, combined with limited cooperation and immature breathing patterns, can reduce the uniform distribution of intranasally administered sprays [35,45]. Experimental and modelling studies have shown that intranasal spray deposition in pediatric airways is highly variable and often concentrated in the anterior nasal cavity, with limited penetration into the posterior regions where MC is more active. This is further influenced by administration technique, spray velocity, and actuation force, all of which significantly affect particle deposition patterns [85]. Computational and in vitro models suggest that younger children may experience reduced intranasal drug delivery efficiency compared with older children and adults, primarily due to smaller nasal volumes and altered airflow dynamics [86,87,88]. However, direct randomized controlled trials comparing the clinical efficacy of nasal sprays across age groups are lacking, and current evidence is therefore indirect based mainly on anatomical and physical modelling rather than clinical endpoints. Despite these limitations, nasal sprays remain widely used in this age group due to their ease of administration and good tolerability, although their clinical impact may be less pronounced than that of high-volume irrigation techniques in conditions characterized by significant secretion burden [62]. The future of NI in children is framed by the growing recognition of non-pharmacological strategies to promote more appropriate and rational use of medical therapies. In pediatric care, the persistent issue of inappropriate antibiotic and decongestant use remains a relevant concern. In this context, saline NI is a safe and effective adjunctive measure that supports mucociliary defense mechanisms and may reduce unnecessary pharmacological treatments [21]. A possible preventive role is increasingly discussed, although it remains to be confirmed by prospective controlled studies. Regular use has been proposed as a potential strategy to reduce the incidence and severity of respiratory episodes, although prospective studies are still needed [21,89]. A key determinant for successful clinical implementation is treatment adherence, which can be substantially improved through early introduction of the technique, progressive familiarization, and active caregiver involvement supported by structured educational interventions. In this regard, major international guidelines, including EPOS and ARIA, as well as recommendations from pediatric and otolaryngology societies, consistently recognize NI as a first-line or adjunctive therapy in common pediatric upper respiratory conditions, given its favorable safety and efficacy profile [90,91]. From a technological perspective, innovation in delivery systems may further enhance clinical effectiveness. The development of ergonomically optimized, pressure-controlled devices, as well as novel formulations with potential anti-biofilm or immunomodulatory properties, could expand the therapeutic role of NI [92,93]. Furthermore, advances in understanding the nasal microbiota and local inflammatory pathways may support the development of more personalized approaches tailored to specific disease phenotypes [94,95]. The integration of robust clinical evidence, technological progress, and structured educational strategies may transform NI from a predominantly empirical intervention into a standardized, evidence-based component of pediatric respiratory care pathways.

## 6. Conclusions

**Figure 1:**
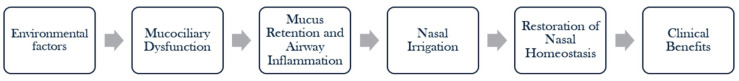
Pathophysiological rationale for nasal irrigation in pediatric upper airway diseases.

NI appears to be a safe and generally well-tolerated intervention across different age groups, including children, with evidence suggesting its potential clinical benefits in selected upper airway conditions. This statement is supported by the pathophysiological rationale of the enhancement of MC and reduction in local microbial burden. By hydrating the periciliary layer, facilitating mechanical removal of secretions and inhaled particles, and potentially modulating mucosal edema and inflammatory mediators, it helps maintain optimal conditions in the upper airway mucosa, thereby improving respiratory comfort and potentially reducing the risk of complications (Figure 1).

Although the available clinical evidence is limited by methodological heterogeneity, it offers preliminary support for the efficacy of NI in common pediatric conditions, including acute URTIs, rhinosinusitis, and AR [96]. Its role as an adjunctive therapy is particularly relevant in children, in whom narrower airways and reduced ability to clear secretions independently make supportive mechanical interventions a reasonable supportive option [39,97] (Table 1).

The clinical impact of NI is particularly relevant in children, in whom narrower airways and reduced ability to clear secretions independently make supportive mechanical interventions a reasonable supportive option [39] (Table 1). The effectiveness of NI depends closely on proper technique, appropriate selection of isotonic or hypertonic solutions, and the use of age-appropriate, safe-delivery devices. Caregiver education and individual tailoring of the procedure are essential to optimize both efficacy and tolerability. When properly performed, NI is a simple, low-cost intervention with a favorable safety profile and minimal adverse effects, making it suitable for integration into pediatric respiratory care. Future research should focus on well-designed randomized controlled trials with standardized protocols and clinically relevant outcomes, to better define optimal parameters such as solution concentration, volume, and frequency of administration. Given the generally low quality of evidence regarding the clinical effectiveness of different nasal irrigation formulations and delivery methods, current data provide only preliminary support for NI as a safe adjunctive intervention for several pediatric upper airway disorders [96]. Further high-quality, standardized randomized controlled trials are strictly needed to firmly define optimal protocols and clarify the comparative superiority of specific solutions and devices [96].

## Figures and Tables

**Table 1:** Main studies about NI in pediatric age.

Author(s)	Year	Study Characteristics	Study Type	Main Research Results	Study Limitations
Garavello et al. [98]	2005	20 children (6–12 years) with Parietaria allergy; compared 3% HS administered via syringe three times daily versus no treatment for 6 weeks.	RCT	Hypertonic nasal rinsing reduced symptoms of seasonal allergic rhinoconjunctivitis and oral antihistamine consumption (*p* < 0.05).	Study was non-blinded and lacked a placebo group. Small sample size was not calculated a priori.
Li et al. [99]	2009	26 children (8–15 years) with persistent AR; compared budesonide only, 0.9% saline only, and combination therapy for 12 weeks.	RCT	Saline irrigation facilitates control of AR and allows for a lower dosage of topical steroids.	Very small sample size (n = 26). The exact mechanism of action remains controversial.
Satdhabudha et al. [100]	2017	80 children (3–15 years) with acute sinusitis; used 1.25% buffered HS twice daily for 2 weeks with two positive-pressure devices.	RCT	Squeezable bottles were superior to syringes in clinical efficacy (5S-score) and patient satisfaction (*p* < 0.05).	Short follow-up period (2 weeks). Lack of a control group receiving no irrigation. High rate (80%) of bacterial device contamination.
Köksal et al. [101]	2016	109 children (<2 years) with acute URTI; compared saline drops, seawater drops, and a control group (no drops) over 7 days.	RCT (Double-blind)	Both saline (0.9%) and seawater (2.3%) significantly improved nasal congestion compared to the control group in infants with the common cold (*p* < 0.05).	Follow-up limited to 7 days. Significant loss to follow-up (16 patients not reached by telephone).
Montanari et al. [102]	2010	435 children (2 months–2 years); prospective observation over 5 months during the cold season.	Observational Study	The Narhinel method (aspirator + saline) reduced episodes of acute otitis media and acute rhinosinusitis (ARS) compared to saline alone.	Non-randomized design; potential bias as pediatricians prescribed routine treatments at their discretion.
Garavello et al. [103]	2003	20 children (6–12 years) with Parietaria allergy; compared 3x daily 3% HS via syringe vs. no treatment for 6 weeks.	RCT	Hypersaline NI (3%) effectively prevented symptoms in children with seasonal AR (*p* < 0.05).	Very small sample size (n = 20). Prospective non-blinded trial with no placebo.
Hong et al. [104]	2014	77 children (≤13 years) with refractory chronic rhinosinusitis; evaluated compliance and outcomes over a mean of 6.2 months.	Retrospective Cohort	NI is effective and well-tolerated (63.6%); good compliance correlated with a lower need for surgery (*p* = 0.019).	Retrospective design. Selection bias, as patients were already refractory to standard medical treatments.
Tugrul et al. [105]	2014	105 children (5–18 years) with Acute Bacterial Rhinosinusitis; compared standard antibiotic therapy (14 days) vs. 3 weeks of large-volume saline + topical steroid.	RCT	The combination of large-volume saline and fluticasone propionate is safe and effective for treating pediatric Acute Bacterial Rhinosinusitis (*p* < 0.05).	Lack of a placebo control group.
Ragab et al. [106]	2015	62 children with uncomplicated ARS; compared 0.9% saline + amoxicillin (100 mg/kg/d) vs. saline + placebo for 14 days.	RCT (Triple-blind)	Isotonic NI alone demonstrated clinical and bacteriological efficacy equivalent to amoxicillin in treating ARS (*p* < 0.05).	Follow-up limited to 14 days. Ethical constraints prevented post-treatment CT scan radiological evaluations.
Alexandrino et al. [107]	2018	44 children (<3 years) with URTI; assessed immediate effects of a single session vs. 30 min of normal activities.	RCT	The rhino-pharyngeal clearance protocol immediately improved nasal obstruction and middle ear peak pressure.	Assessed only immediate effects. High data loss (17 patients) due to corrupted audio recordings of nasal sounds.
Khoshdel et al. [108]	2014	80 children (4–15 years) with ARS; compared 14 days of amoxicillin + 5 days irrigation vs. irrigation alone.	RCT (Double-blind)	Adding amoxicillin to NI provided limited therapeutic benefits compared to saline irrigation alone by day 14 (*p* < 0.05).	Irrigation duration was limited to 5 days. 20% dropout rate during the study.
Wei et al. [109]	2011	40 children (4–17 years) with Chronic Rhinosinusitis; 6-week course of once-daily 45 mL saline vs. saline + 80 mg gentamicin.	RCT (Double-blind)	Isotonic saline and saline + gentamicin were equally effective in improving quality of life (and computer tomography findings in pediatric Chronic Rhinosinusitis).	Small sample size (n = 34 completed). Topical antibiotics may have limited reach into sinuses through narrow natural ostia in unoperated patients.
Pham et al. [110]	2014	104 children (<18 years) with Chronic Rhinosinusitis; retrospective analysis of computed tomografy outcomes and long-term parental follow-up survey (median 48 months).	Retrospective Cohort	Daily NI significantly reduced radiological scores (*p* < 0.05) and the long-term need for functional endoscopic sinus surgery.	Recall bias in the parental surveys. Single clinic setting; prospective quality of life surveys were not collected for the entire cohort.
Cho et al. [111]	2016	26 children (5–18 years) with Chronic Rhinosinusitis; compared 4 weeks of twice-daily HOCl vs. isotonic saline irrigation.	RCT	Low-concentration hypochlorous acid irrigation was more effective than isotonic saline in improving radiographic (X-ray) parameters (*p* = 0.023).	Very small sample size (n = 26 analyzed). Used standard X-ray instead of CT to minimize radiation exposure.
Shoseyov et al. [112]	1998	30 children (3–16 years) with chronic maxillary sinusitis: 4 weeks of 3.5% HS versus 0.9% saline drops three times dai-ly.	RCT (Double-blind)	Hypersaline nasal (3.5%) was superior to normal saline (0.9%) in reducing cough and radiological findings in chronic sinusitis (*p* < 0.05).	Extremely small sample size (n = 30). Potential tolerability issues, with 4 children dropping out due to a burning sensation.

## Data Availability

No new data were created or analyzed in this study.

## Abbreviations

The following abbreviations are used in this manuscript:

NI	Nasal Irrigation
MC	Mucociliary Clearance
URTI/URTIs	Upper Respiratory Tract Infection(s)
CT	Computed Tomography
ET	Eustachian tube
HS	Hypertonic solution
PM2.5/PM10	Particulate Matter
AR	Allergic Rhinitis
ARS	Acute Rhinosinusitis
RCT	Randomized Controlled Trial
ARIA	Allergic Rhinitis and its Impact on Asthma
EPOS	European Position Paper on Rhinosinusitis and Nasal Polyps
